# RAD001 targeted HUVECs reverses 12‐lipoxygenase‐induced angiogenesis in oesophageal squamous cell carcinoma

**DOI:** 10.1111/jcmm.16705

**Published:** 2021-06-13

**Authors:** Xue Chen, Xuan Chen, Xiaozheng Sun, Cong Wang, Zhihua Wen, Yufeng Cheng

**Affiliations:** ^1^ Departments of Radiation Oncology Qilu Hospital of Shandong University Jinan China

**Keywords:** 12‐lipoxygenase, angiogenesis, ESCC, mTOR, RAD001

## Abstract

12‐LOX plays an important role in the progression of various malignancies. However, the underlying mechanisms of the action of 12‐LOX and tumour treatment strategies remain not fully defined. In this study, we investigated the possible roles of 12‐LOX in ESCC and explored the new therapeutic target. Approximately 73% of ESCC tissues showed marked up‐regulation of 12‐LOX, which was associated with poor prognosis. 12‐LOX overexpression was positively correlated with the malignant progression of ESCC as demonstrated both in vitro and in vivo. Up‐regulation of 12‐LOX significantly increased the proliferation of ESCC cells and the xenograft volume. Moreover, 12‐LOX up‐regulation promoted tube formation of HUVECs and tumour angiogenesis in xenografts. Mechanism investigation indicated that 12‐LOX overexpression led to activation of the PI3K/AKT/mTOR pathway and the up‐regulation of VEGF in ESCC cells. Subsequent analysis indicated that the RAD001 could reverse the 12‐LOX‐induced promoting effect on ESCC. Specifically, the application of RAD001 inhibited the proliferation of ESCC cells and the tube‐forming ability of HUVECs. In the drug group, the xenografts exhibited significant volume reduction and angiogenesis inhibition. We demonstrated that RAD001 could inhibit HUVEC migration. These findings presented the evidence that RAD001 had distinct roles on HUVECs and could exert anti‐tumour effects by targeting not only the PI3K/AKT/mTOR pathway but the angiogenesis in ESCC.

## 1 INTRODUCTION

Oesophageal cancer (EC) is a common malignancy that is associated with poor prognosis and leads to high mortality rate. It is the eighth most common cancer worldwide and the sixth leading cause of cancer‐related deaths.^1^ The two main pathological types of EC are squamous cell carcinoma (ESCC) and adenocarcinoma (EAC). In the highest risk area, 90% of cases are squamous cell carcinoma.^2^ EC is the third most common cancer in China after lung and stomach cancer. China accounts for approximately 50% of the total EC cases worldwide.^2^, ^3^ An epidemiological survey of malignant tumours in China in 2015 indicated that the annual incidence of EC was 477 900/1.37 billion, and there were 375 000 deaths every year.^4^ Due to the atypical early symptoms of EC and the lack of specific tumour markers for early diagnosis, the majority of the patients are diagnosed with an advanced stage and cannot be actively treated, resulting in poor prognosis. The 5‐year survival rate is only 15% to 25%.^5^, ^6^ Therefore, there is an urgent need to explore biomarkers for early diagnosis of ESCC and the treatment strategies to improve patient prognosis.

Arachidonic acid (AA) is a long‐chain polyunsaturated fatty acid (PUFA) widely distributed in mammalian cell membranes. The lipoxygenase (LOX) pathway is the main pathway of AA metabolism, as well as the cyclooxygenase (COX) and the cytochrome P450 (CYP450) pathways. The metabolites of AA obtained through these pathways are collectively called eicosanoids, which are essential components of the cell membrane, participating in cell membrane function and cell signalling transduction. The dysregulation of bioactive lipids, especially LOX and its metabolites, is implicated in a variety of pathophysiological processes and diseases, including inflammation and cancers. It is known that LOX and HETE can promote the progression of various human malignant tumours, and the mechanisms include the inhibition of apoptosis,^7^ induction of cell proliferation^7^, ^8^, ^9^ and metastasis of cancer^10^, ^11^, ^12^ cells and activation of tumour angiogenesis.^13^, ^14^

Tumorigenesis is often driven by maladjustment of cellular information processing and signal transduction. As normal cells evolve progressively to a neoplastic state, they acquire a succession of hallmark capabilities that contribute to tumorigenesis. Inducing angiogenesis and maintaining proliferative signals are important signs of cancer.^15^ Tumour microvascular structure has long been viewed as a core direction of tumour research. Tumour microvasculature is a crucial compartment in TME, which plays a significant role in tumour energy and oxygen supply and tumour metabolism. During tumour development, persistent pro‐angiogenic signals lead to the continued growth of new blood vessels in the quiescent vascular system, which in turn helps maintain tumour growth. However, tumour neovascularization caused by chronic stimulation and unbalanced pro‐angiogenic signals is usually abnormal. Compared with normal blood vessels, tumour blood vessels show tortuous and disordered structures, uneven thickness, arteriovenous and vascular malformation and incomplete tube wall formation. This aggravates the hypoxic environment of the tumour, making tumour cells less responsive to chemoradiotherapy and eventually leading to chemoradiotherapy resistance.^16^ In addition, abnormal blood vessels may promote appropriate conditions for tumour metastasis. Therefore, the investigation of tumour microvasculature is of great significance for understanding the process of tumour development, metastasis and drug resistance, as well as the prevention, treatment and prognosis of cancer‐related diseases.

The phosphatidylinositol 3‐kinase (PI3K)/AKT/mTOR pathway (mammalian target of rapamycin) is activated in most human cancers,^17^, ^18^, ^19^ which plays an important role in numerous cellular functions including metabolism, proliferation, adhesion, migration, invasion, survival and angiogenesis.^20^, ^21^ Therefore, the PI3K/AKT/mTOR pathway is an important target for cancer treatment, and several inhibitors of this pathway have been developed.^22^, ^23^ The mTOR pathway plays an important role in regulating cell metabolism, proliferation and survival in response to environmental signals.^24^, ^25^, ^26^ Accumulated clinical evidence has shown that the mTOR inhibitors exhibit effective suppression effect against malignant tumours.^27^ Everolimus (RAD001) is the most commonly used mTOR inhibitor, which directly inhibits mTORC1, but has limited or no effect on mTORC2 activity.^28^ Currently, everolimus is the only mTOR inhibitor approved by the Food and Drug Administration (FDA) for the treatment of certain types of breast cancer, pancreatic neuroendocrine tumours and papillary renal carcinoma. RAD001 has been shown to exhibit anti‐tumour effects in several cancers by inhibiting tumour growth and reducing tumour vascularization.^29^, ^30^, ^31^

## 2 MATERIALS AND METHODS

### 2.1 Patients and tissue samples

Paraffin sections of tumour tissues and adjacent tissues were obtained from 153 patients with pathologically confirmed diagnosis of ESCC at Qilu Hospital of Shandong University and Linyi County People's Hospital from February 2010 to December 2011. All patients were followed up for at least 3 years, and their clinicopathological features were recorded.

### 2.2 Cell culture

Human ESCC cell lines Kyse150, Eca109, Eca9706 and TE‐1 were purchased from the cell bank of the Chinese Academy of Sciences and cultured in RPMI‑1640 medium (Gibco; Thermo Fisher Scientific, Inc, Waltham, MA, USA) containing 10% foetal bovine serum (FBS, Gibco; Thermo Fisher Scientific, Inc), streptomycin (100 mg/ml) and penicillin (100 U/ml). All cells were cultured in a humidified incubator at 37℃ with 5% CO_2_.

### 2.3 Lentiviral vector infection

The control and 12‐LOX overexpression lentiviral vectors were constructed by GeneChem (Shanghai, China). Kyse150 cells were seeded in 6‐well plates with a density of 5 × 10^4^ cells/well. 12‐LOX overexpression vectors or control vectors were infected into Kyse150 cells according to the relevant manufacturer's instructions when the confluency reached 30%‐40%, and the medium was replaced at 24 hours after infection. The infected cells were then selected with 3 µg/ml of puromycin to establish cells stably expressing 12‐LOX at 72 hours. After 2 passages, the cells were used for subsequent experiments and divided into two groups as follows: cells infected with control vector (LV‐Ctrl group) and 12‐LOX overexpression vector (LV‐12‐LOX group).

### 2.4 Colony formation assay

Cells were seeded in 6‐well plates (800 cells/well). The adherent 12‐LOX‐Kyse150 cells were treated with gradient concentration inhibitors (LY294002 and RAD001, Selleckchem; Houston, TX, USA) or control DMSO for two weeks prior to staining. The colonies were fixed in methanol and stained with crystal violet, and then, the number of colonies was measured by counting more than 50 cells (>50 cells was defined as a colony) (ImageJ 1.47v software; NIH; National Institutes of Health, Bethesda, MD, USA).

### 2.5 Cell Counting Kit‐8 (CCK‐8) assay

Cell viability was assessed using the Cell Counting Kit‐8 (CCK‐8, BestBio; Shanghai, China). Ctrl‐Kyse150 and 12‐LOX‐Kyse150 cells were plated in 96‐well plates (1000 cells/well). A total of 100 μl culture medium containing gradient concentration inhibitor was added to each well. Subsequently, the viability of each group of cells was assessed at 24, 48, 72 and 96 hours by measuring 450 nm absorbance using a microplate reader (Thermo Scientific, Inc, Vantaa, Finland).

### 2.6 EDU proliferation assay

Ctrl‐Kyse150 and 12‐LOX‐Kyse150 cells were plated into 24‐well plates (4 × 10^4^ cells/well) for cell climbing. Following adherence of the cells, they were treated with LY294002 or RAD001 for 48 hours in RPMI‑1640 with 10% FBS and then stained with EdU using EdU incorporation assay kit (Ribobio; Guangzhou, China) according to the manufacturer's instructions. Five fields from each cell climbing well were randomly captured with Olympus BX53 DP72, and three independent experiments were performed. The number of EdU‐positive cells was calculated with ImageJ 1.47V.

### 2.7 Tube formation assay

HUVEC cells were pre‐incubated with conditioned medium for 24 hours prior to seeding into the 96‐well plates. The conditioned medium (containing various concentrations of LY294002 and RAD001) was composed of a 1:1 mixture of cell culture supernatant and DMEM. The 96‐well plates were coated with 50 μl Matrigel (BD Biosciences; Franklin Lakes, NJ, USA) according to the manufacturer's instructions and incubated at 37℃ for 30 min. Subsequently, HUVECs were seeded on coated plates (4 × 10^4^ cells/well) and cultured with serum‐free medium at 37℃ for 4 hours. Tube formation was observed and photographed using an Olympus DP71 microscope. Five random fields were selected in each well, and the total tube length was quantified using the NIH ImageJ 1.47v software. Each group was tested in triplicate.

### 2.8 Transwell assay

HUVEC cells were seeded into 6‐well plates. After RAD001 stimulation for 24 hours, cells were trypsinized, counted and plated into a transwell chamber (pore size: 8 μm, 24‐well) with serum‐free medium (5 × 10^4^ cells/well). 20% FBS‐containing medium was used to induce cell migration. The migrating cells were fixed after 12 hours, stained with crystal violet, photographed and counted.

### 2.9 Western blot analysis

Cells were seeded into 6‐well plates. Following overnight incubation, the cells were treated with LY294002, RAD001 or DMSO and harvested at 48 hours. Western blot analysis was performed as previously described.^32^ The membranes were exposed with an enhanced chemiluminescence reaction (ECL) kit, and the grey levels of the protein bands were analysed using the ImageJ 1.47v software. The sources of the primary antibodies applied were as follows: GAPDH (#2118), β‐tubulin(#2146), p‐PI3K S1981 (#4228), PI3K (#4249), p‐AKT S473(#4060), AKT(#4685), p‐mTOR S2448 (#5536), mTOR (#2983), p‐P70S6KT421/S424 (#9204), P70S6K(#2708), p‐S6 S235/236 (#4858) and S6 (#2217) were purchased from Cell Signaling Technologies; polyclonal rabbit anti‐12‐LOX was from Novus Biologicals (NBP2‐29941; Novus Biologicals; Centennial, CO, USA); VEGF antibody was obtained from Santa Cruz (sc‐7269; Santa Cruz; Dallas, TX, USA).

### 2.10 Immunohistochemical/Immunofluorescence analysis

Immunohistochemical staining and qualitative scoring were performed as previously described.^32^ All antibodies applied were validated by immunohistochemistry (IHC) and immunofluorescence (IF) in paraffin‐embedded tissues as determined by the manufacturer. The average fluorescence intensity was measured with ImageJ 1.47V. The following primary and secondary antibodies were used for immunohistochemistry/immunofluorescence (IHC/IF): phospho‐mTOR (Ser2448) antibody (#2976; Cell Signaling Technology; Danvers, MA, USA); CD31 antibody (#3528); 12‐lipoxygenase antibody (NBP2‐29941; Novus Biologicals; Centennial, CO, USA); Andy Fluor™ 488 Goat Anti‐Rabbit IgG (H+L) antibody (L110A; GeneCopoeia; Rockville, MD, USA); and Andy Fluor™ 594 Goat Anti‐Mouse IgG (H+L) antibody(L119A).

### 2.11 In vivo experiments

All animal experiments were approved by the Ethics Committee of Qilu Hospital of Shandong University and were carried out in accordance with the national regulations for animal research in China. Female BALB/c nude mice aged 4 weeks were randomly divided into 2 groups and maintained under standard conditions according to institutional guidelines for animal care. LV‐Ctrl‐Kyse150 or LV‐12‐LOX‐Kyse150 cells (1 × 10^6^) were suspended in PBS and injected subcutaneously into the flank of the mice. RAD001 intervention (diluted with NS, intraperitoneal injection, 3mg/kg) was performed once the tumours had reached a volume of 100 mm^3^, and the compound was administered daily for 7 days. Tumour size was measured every five days using callipers and the tumour volumes were calculated using the following equation: V = (length × width^2^)/2. The mice were killed at the end of the study, and the tumours were dissected four weeks after appearance and stored at −80℃ for subsequent analysis. The flow chart showed the entire process of the in vivo experiment (Figure 6A).

### 2.12 Statistical analysis

Unpaired t test was conducted using the SPSS software 24.0 (IBM Corp., Armonk, NY, USA). Kaplan‐Meier method and log‐rank test were used for survival analysis. The results were presented as mean ±standard error (SE). *P* value <0.05 was considered a significant difference.

## 3 RESULTS

### 3.1 12‐LOX overexpression was associated with poor prognosis of ESCC

**FIGURE 1:**
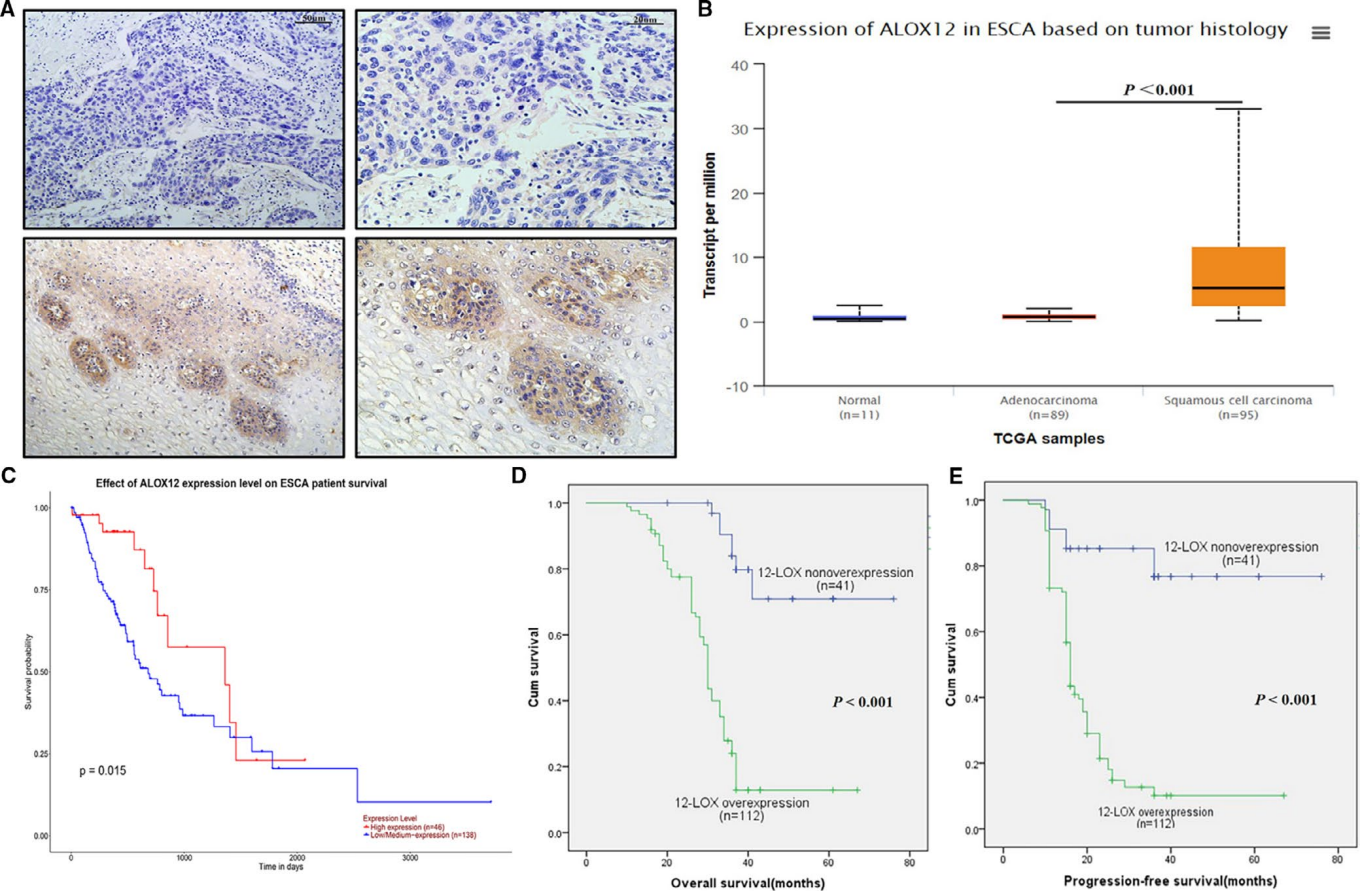
12‐LOX(ALOX12) is up‐regulated in ESCC tissues and associated with poor prognosis of ESCC patients. A, Representative negative (upper) and positive (below) 12‐LOX IHC staining. Brown represents positive staining. Scale bar = 20 or 50 µm. B, 12‐LOX mRNA expression data in different histological types acquired from TCGA. C, Relationship between 12‐LOX expression and prognosis data acquired from TCGA. D, Kaplan‐Meier curve for OS. E, Kaplan‐Meier curve for PFS. 12‐LOX, lipoxygenase; ESCC, oesophageal squamous cell carcinoma; ESCA, oesophageal carcinoma; IHC, immunohistochemistry; TCGA, The Cancer Genome Atlas; OS, overall survival; PFS, progression‐free survival. Data are presented as the mean ±SEM. *P < 0.05; **P < 0.01; ***P < 0.001

IHC detection was conducted in all 153 ESCC cases (see Table S1 for the basic characteristics) to assess the expression levels of 12‐LOX. The results indicated that 112 cases were included in the 12‐LOX high‑expression group (score >8) and 41 cases in the 12‐LOX low‐expression group (score ≤8). IHC staining results of 12‐LOX was shown in Figure 1A. TCGA database mining indicated that the expression level of 12‐LOX (ALOX12) exhibited no significant correlation with the parameters of gender, age, smoking and alcohol consumption habit of ESCC patients, whereas a strong correlation was noted with histological type *(* http://ualcan.path.uab.edu/cgi‐bin/TCGAExResultNew2.pl?genenam=ALOX12&ctype=ESCA *)*. The expression level of 12‐LOX in squamous cell carcinoma was significantly higher than that in normal oesophageal and adenocarcinoma tissues (Figure 1B). The survival outcome of ESCC patients was consistent with TCGA data (Figure 1C; *P* = 0.015). The OS and PFS of the patients in the 12‐LOX high‐expression group were significantly lower than those in the low‐expression group (Figure 1D, E, *P* < 0.001, respectively).

### 3.2 12‐LOX promoted proliferation of ESCC cells

**FIGURE 2:**
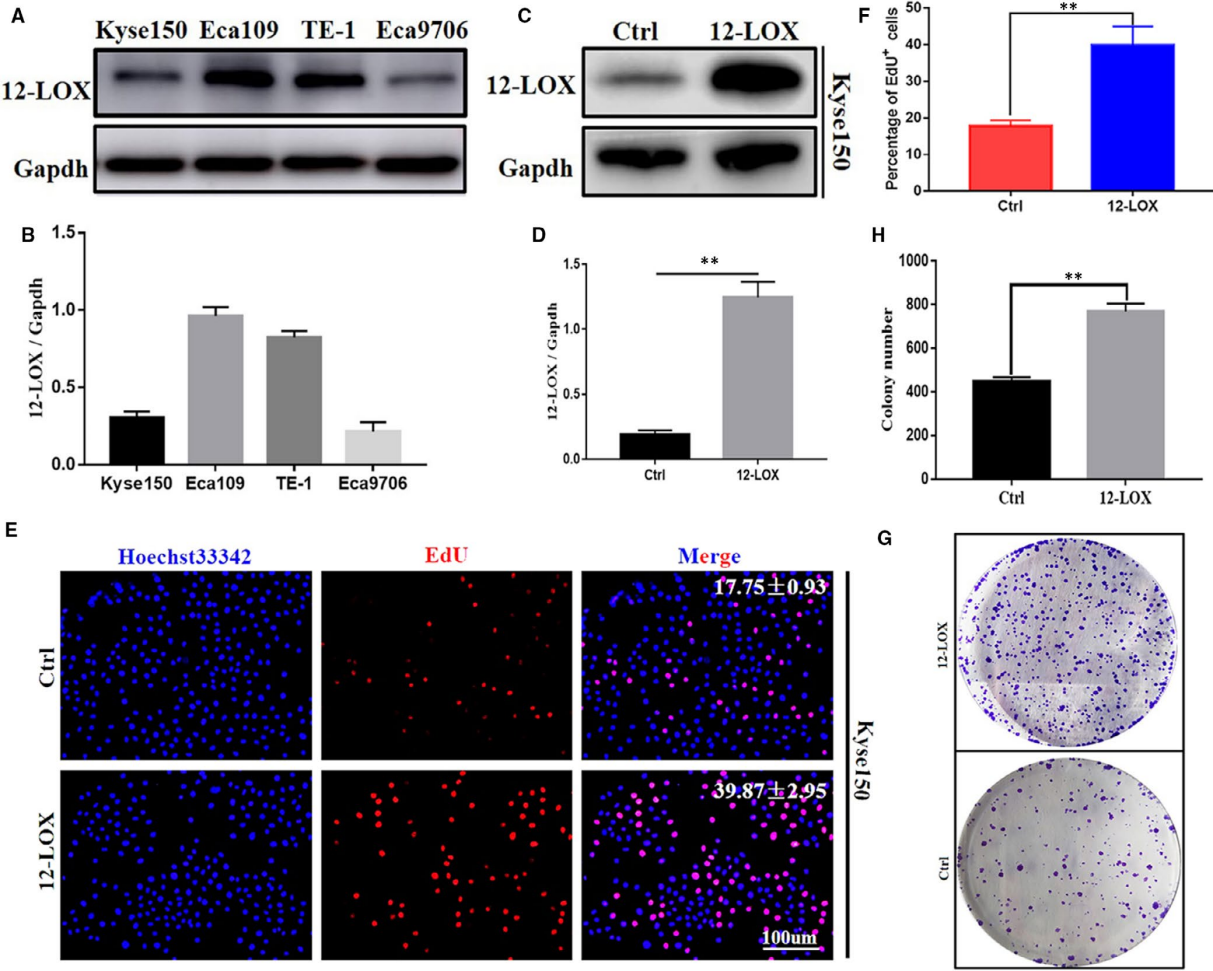
12‐LOX promoted the proliferation ability of ESCC Kyse150 cells in vitro. A, B, The basic expression of 12‐LOX in different ESCC lines. C, D, The up‐regulation efficiency of 12‐LOX in Kyse150 cells. E, F, EdU assays for Kyse150‐LV‐Ctrl and Kyse150‐LV‐12‐LOX cells and quantification of EdU‐positive cells. The mitotic cells were labelled with EdU (red), and nucleus was labelled with Hoechst 3334 (blue), and images were merged. Scale bar = 100 μm. G, H, Representative images of colony formation for Kyse150‐LV‐Ctrl and Kyse150‐LV‐12‐LOX cells and quantification of colonies. 12‐LOX, lipoxygenase; ESCC, oesophageal squamous cell carcinoma; EdU, 5‐Ethynyl‐2'‐deoxyuridine. Data are presented as the mean ±SEM. *P < 0.05; **P < 0.01; ***P < 0.001

Th**e** basal expression of 12‐LOX in Eca109 and TE‐1 cells was higher than that in Kyse150 and Eca9706 cells (Figure 2A, B). 12‐LOX was overexpressed in Kyse150 cell line for subsequent analysis, and the overexpression efficiency was verified accordingly (Figure 2C, D).

To explore the role of 12‐LOX in ESCC cell proliferation, EdU, colony formation and the CCK‐8 assays were conducted. The EdU assay indicated that the 12‐LOX overexpression group exhibited higher percentage of EdU‐positive cells than the control group (Figure 2E, F). The colony formation assay showed higher number of clones in the 12‐LOX overexpression group (Figure 2G, H) compared to the control group. The CCK‐8 assay suggested that the OD value of the LV‐12‐LOX group was significantly higher than that of the LV‐Ctrl groups at 24, 48 and 72 hours (Figure S1). Colony formation assay showed that Eca109 (relatively higher expression of 12‐LOX) formed less colonies under the stimulation of Baicalein (40 μM), a selective inhibitor of 12‐LOX (Figure S2). All these results suggested that high expression of 12‐LOX led to a more potent cell proliferative capacity in ESCC.

### 3.3 12‐LOX promoted migration of HUVECs and tube formation

**FIGURE 3:**
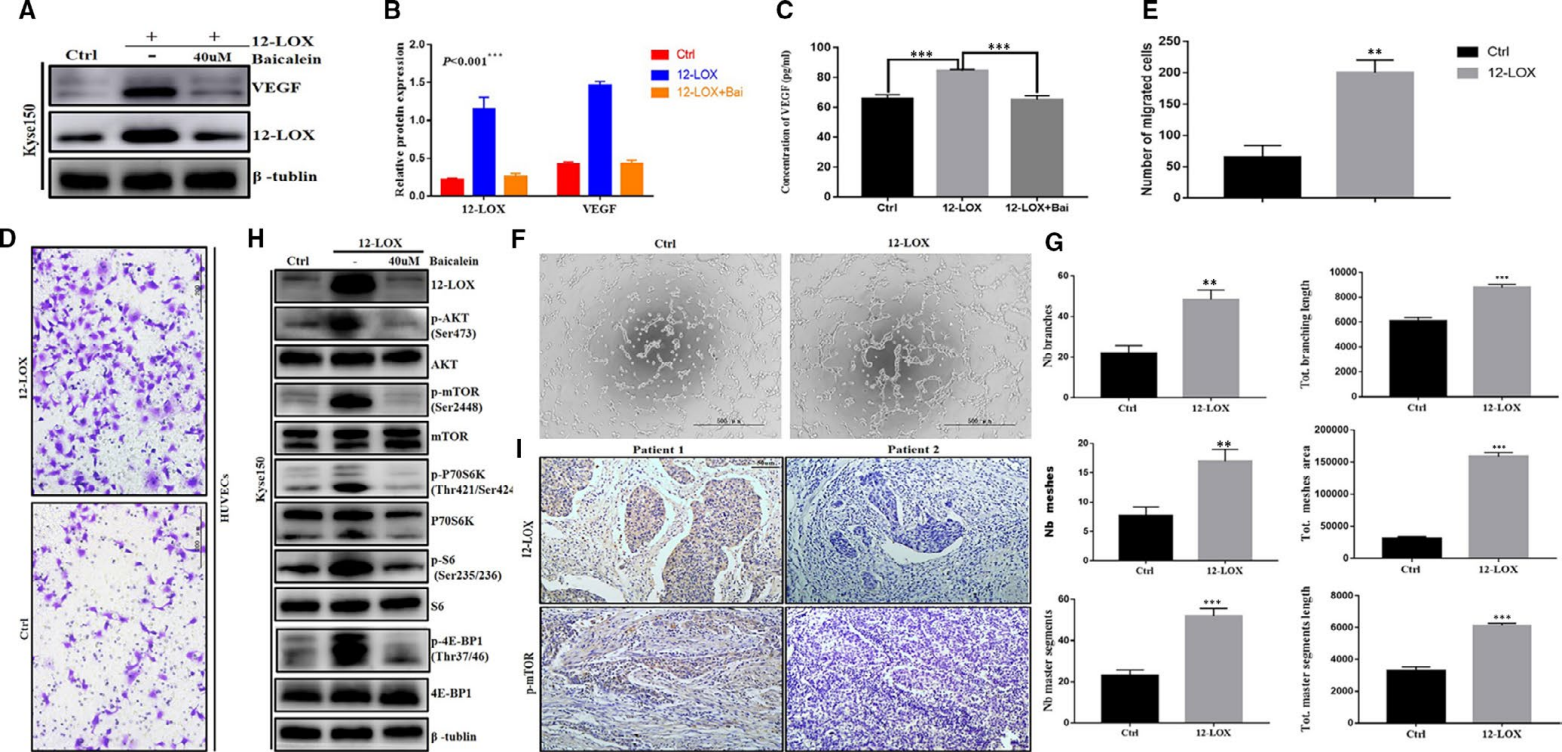
12‐LOX(ALOX12) up‐regulation enhances angiogenesis and activates the PI3K/AKT/mTOR pathway in vitro. Baicalein, a selective inhibitor of 12‐LOX. A, B, Western blotting of VEGF in different treated Kyse150 cells groups indicated. C, ELISA of VEGF in the supernatants of different treated Kyse150 cells. D, E, Transwell assay of HUVECs under control and conditioned medium after incubation for 6h. Scale bar = 200 µm. F, Tube formation of HUVECs under control and conditioned medium. Scale bar = 500 µm. G, The branch, mesh and master segments statistics of tube formation. H, Immunoblots of 12‐LOX, phosphorylated proteins of PI3K/AKT/mTOR pathway. I, Staining for p‐mTOR performed in human samples. 12‐LOX, lipoxygenase; ESCC, oesophageal squamous cell carcinoma; HUVECs, human umbilical vein endothelial cells. Data are presented as the mean ± SEM. *P < 0.05; **P < 0.01; ***P < 0.001

Previous studies have shown that lipoxygenase is an important factor required for VEGF‐mediated angiogenesis.^33^, ^34^ Therefore, the expression of VEGF in ESCC cells was assessed. Western blot analysis (Figure 3A, B) and ELISA (Figure 3C) were used to detect the content of VEGF. The results indicated that VEGF level in the 12‐LOX overexpression group was significantly higher than that in the control group.

Migration of endothelial cells is another key step in angiogenesis, which allows cells to expand from existing vessels. Subsequently, it was demonstrated that 12‐LOX could promote cell migration and tube formation of HUVECs, which could be used to establish a model to assess endothelial function and angiogenesis. As expected, conditioned medium used in LV‐12‐LOX group significantly promoted HUVECs migration and tube formation compared with the control group (Figure 3D‐G). As shown in Figure 3D, E, conditioned medium significantly increased the number of migrating HUVEC cells in LV‐12‐LOX group compared with the control cells. Moreover, following stimulation under the conditioned medium, tube formation of LV‐12‐LOX group was highly increased compared with that of the control group (Figure 3F). The conditioned medium led to a significant advantage of mesh, master segment and branch in tubes (Figure 3G). Specifically, the number and length of mesh, master segment and branch in the 12‐LOX overexpression group was higher than those in the control group (*P* < 0.001, respectively). Overall, these results indicated that 12‐LOX may promote angiogenesis in vitro by accelerating endothelial cell migration and tubular structure formation.

### 3.4 Overexpression of 12‐LOX activated the PI3K‐AKT‐mTOR pathway

In order to explore the intrinsic biological function of 12‐LOX in ESCC, we further examined the PI3K‐AKT‐mTOR pathway. The results indicated that the phosphorylation levels of AKT and mTOR and of the downstream substrate proteins of the mTOR signalling pathway (P70S6K/S6/4EBP1) were specific activated and increased significantly in 12‐LOX up‐regulated cell lines. And the activation of the pathway was significantly inhibited with the application of Baicalein (Figure 3H). The conclusion was replicated in patients' tissues, and IHC staining showed that patients with high expression of 12‐LOX also had higher mTOR expression (Figure 3I).

### 3.5 12‐LOX exerted a tumour‐promoting effect *in vivo*

**FIGURE 4:**
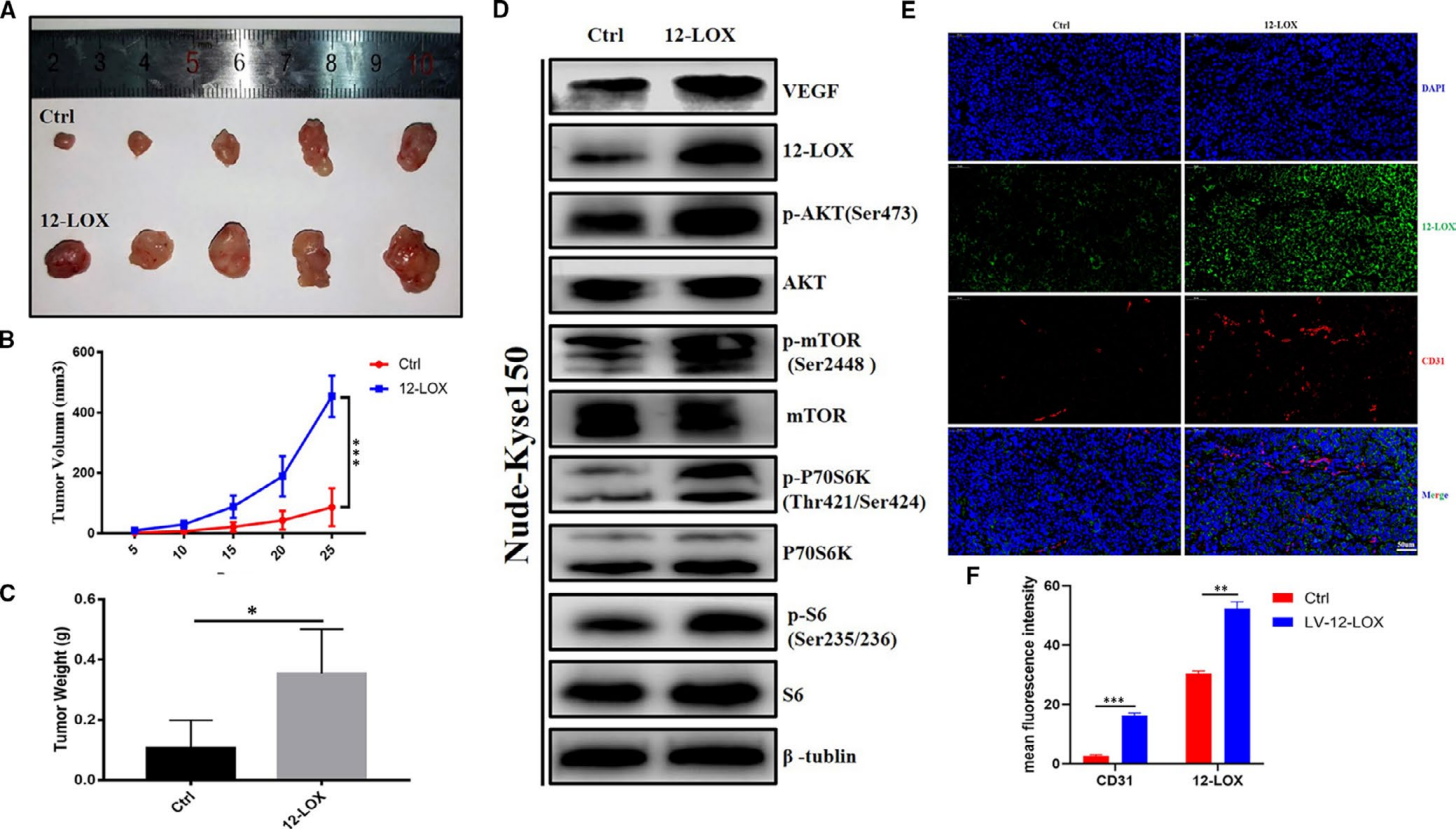
12‐LOX(ALOX12) up‐regulation play a pro‐tumour role in vivo. A, Representative images of subcutaneous Kyse150‐LV‐Ctrl and Kyse150‐LV‐12‐LOX xenografts after surgical removal. B, Tumour growth curves in nude mice of the two groups. C, Tumour weight of the two groups. D, Immunoblots of 12‐LOX, VEGF, phosphorylated proteins of PI3K/AKT/mTOR pathway in vivo. E, Representative images of IF performed on Kyse150‐LV‐Ctrl and Kyse150‐LV‐12‐LOX xenografts with 12‐LOX (green) and CD31 (red) antibodies. Nucleus was labelled with DAPI (blue), and images were merged. Scale bar = 50 µm. F, The expression levels of 12‐LOX and CD31 in 12‐LOX‐overexpressing Kyse150. 12‐LOX, lipoxygenase; ESCC, oesophageal squamous cell carcinoma; IF, immunofluorescence. Data are presented as the mean ±SEM. *P < 0.05; **P < 0.01; ***P < 0.001

To further verify the pro‐tumour effect of 12‐LOX in vivo, a xenograft model of ESCC was established with Kyse150 cells. The increased volume and weight of the tumours implanted subcutaneously in the LV‐12‐LOX group further confirmed the acceleration effect of 12‐LOX on ESCC growth (Figure 4A‐C).

Protein expression levels from xenografts were detected, and the results demonstrated that VEGF, phospho‐AKT, phospho‐mTOR, phosphor‐P70S6K and phosphor‐S6 protein levels in vivo exhibited a consistent trend with in vitro cell results (Figure 4D). The PI3K/AKT/mTOR pathway was activated in the LV‐12‐LOX group. The induction of angiogenesis of the xenograft tumours was detected simultaneously in both groups. IF was performed on paraffin sections of xenografts, and the results demonstrated a positive correlation between 12‐LOX and the vascular endothelial marker CD31. Specifically, the number of blood vessels in the 12‐LOX overexpression group was significantly higher than that in the control group (Figure 4E, F). Overall, the results of these in vivo experiments further demonstrated the tumour‐promoting effect of 12‐LOX on the development of ESCC.

### 3.6 mTOR inhibitor RAD001 could reverse the pro‐tumour effects of 12‐LOX

**FIGURE 5:**
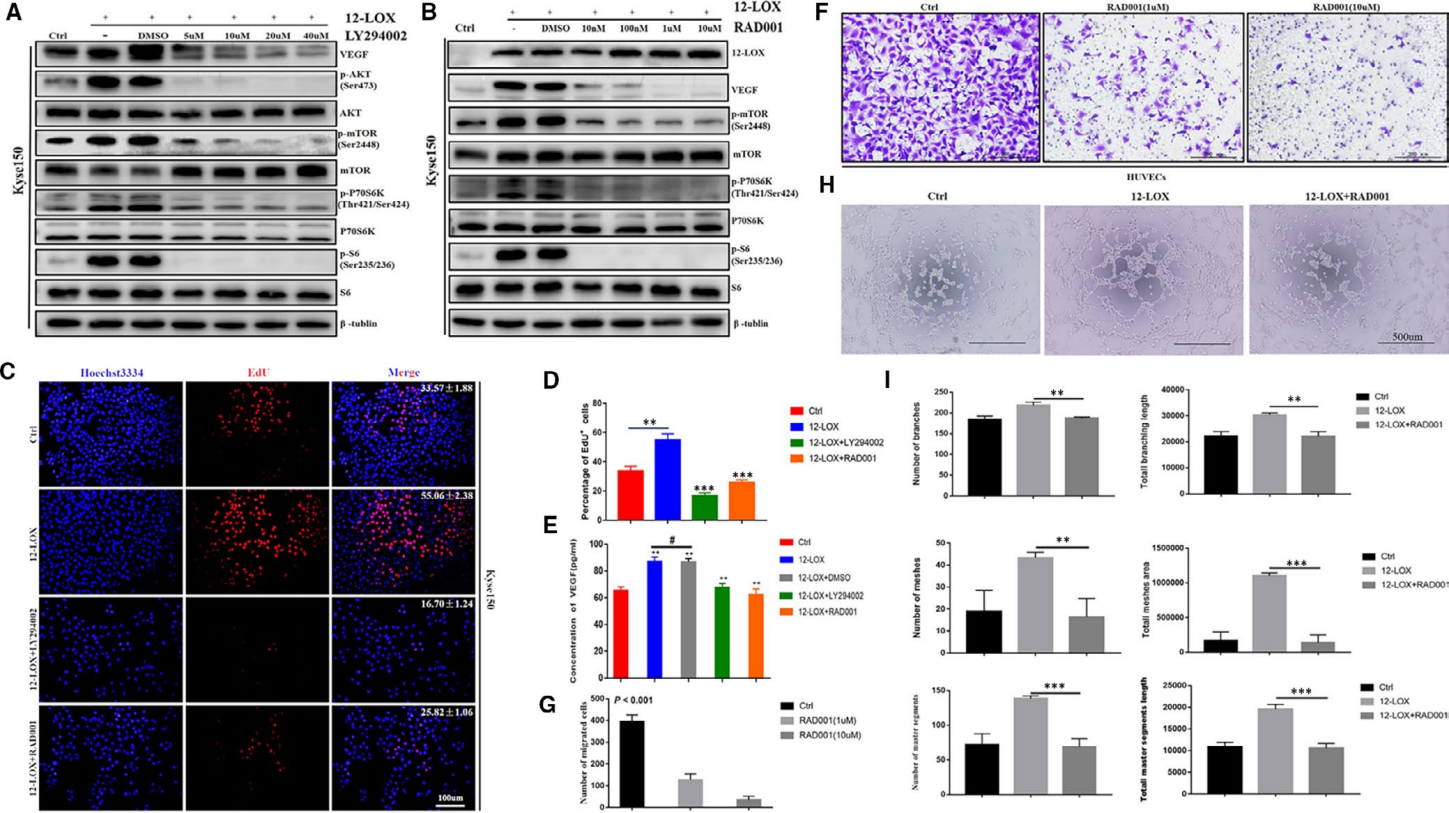
The PI3K/AKT/mTOR pathway inhibitors LY294002 and RAD001 reverse the role of 12‐LOX in promoting angiogenesis and proliferation of ESCC cells. A, B, Immunoblots of VEGF, proteins of PI3K/AKT/mTOR pathway in indicated cells. C, D, Cell proliferation ability under stimulated of LY294002 (20 µm) and RAD001 (1 µm) in EdU (red) assays. Scale bar = 100 μm. E, ELISA of VEGF in the supernatant of different treated Kyse150 cells. F, Images of Transwell assays performed with HUVECs treated with different concentration of RAD001 for 12 h. Scale bar = 200μm. G, Quantification of migrated cells in Transwell assays. H, Representative image of tube formation assay performed with HUVECs under control and conditioned medium. I, The branch, mesh and master segments statistics of tube formation. 12‐LOX, lipoxygenase; ESCC, oesophageal squamous cell carcinoma; IF, immunofluorescence. Data are presented as the mean ± SEM. *P < 0.05; **P < 0.01; ***P < 0.001

A number of inhibitors of the PI3K/AKT/mTOR pathway have been discovered, and these agents have been shown to reduce VEGF secretion and restrain angiogenesis.^35^ To confirm the interaction between the tumour‐promoting effect of 12‐LOX in the development of cancer phenotype and the activation of the PI3K/AKT/mTOR pathway, the latter pathway was suppressed with inhibitors and the cancer‐associated phenotype was further examined. The biologically active doses of the inhibitors in Kyse150 cells were initially established. LY294002 at 20 μM completely abrogated p‐AKT and p‐mTOR activity. RAD001 at 1 μM could inhibit the PI3K/AKT/mTOR pathway and at 10 μM completely abrogated the expression levels of p‐mTOR, p‐P70S6K and p‐S6. Our functional tests were carried out under these concentration stimuli. In addition, the inhibitory effect of LY294002 and RAD001 on VEGF was dose‐dependent (Figure 5A, B).

Subsequently, the proliferation of ESCC cells and the induction of angiogenesis following the use of LY294002 and RAD001 were measured. The proliferation of ESCC cells following treatment with the inhibitors was examined using EdU (Figure 5C, D), CCK‐8 (Figure S3A) and colony formation assays (Figure S3B, C). The number of EdU‐positive cells and colonies was decreased following the treatment of LY294002 and RAD001. The results of the CCK‐8 assay indicated that the OD value of the inhibitor‐treated group was significantly lower than those of the LV‐12‐LOX group at 24, 48, 72 and 96 hours. These data demonstrated that LY294002 and RAD001 treatment led to decreased ESCC cell proliferation.

VEGF detection, HUVEC migration and tube formation assay were used to evaluate the effect of RAD001 on angiogenesis. As expected, VEGF secretion, migratory HUVECs and tube formation were suppressed following inhibitor treatment. According to the results of ELISA (Figure 5E), compared with the control group, RAD001‐treated Kyse150 cells secreted less VEGF. Transwell migration analysis (Figure 5F, G) showed that the number of HUVECs crossing the bottom membrane was decreased in the RAD001‐treated group. Similarly, in the wound healing experiment (Figure S4A, B), the crawling ability of HUVECs stimulated by RAD001 was significantly lower than that of the controls and the creeping distance was shorter at 12, 24 and 48 hours. Consistently, the conditioned medium containing inhibitors reversed the catalytic effect of 12‐LOX on tube formation, which was not noted in the absence of the inhibitors (Figure 5H, I). The conditioned medium containing inhibitors led to a significant suppression of mesh, master segment and branch in HUVECs, which was reflected in the number and length of mesh, master segment and branch. These results indicated that 12‐LOX promoted angiogenesis and cell proliferation in ESCC through the PI3K/AKT/mTOR pathway and that RAD001 might be a potentially powerful cancer‐preventing agent in ESCC.

### 3.7 RAD001 reversed the pro‐tumour effect of 12‐LOX in vivo

**FIGURE 6:**
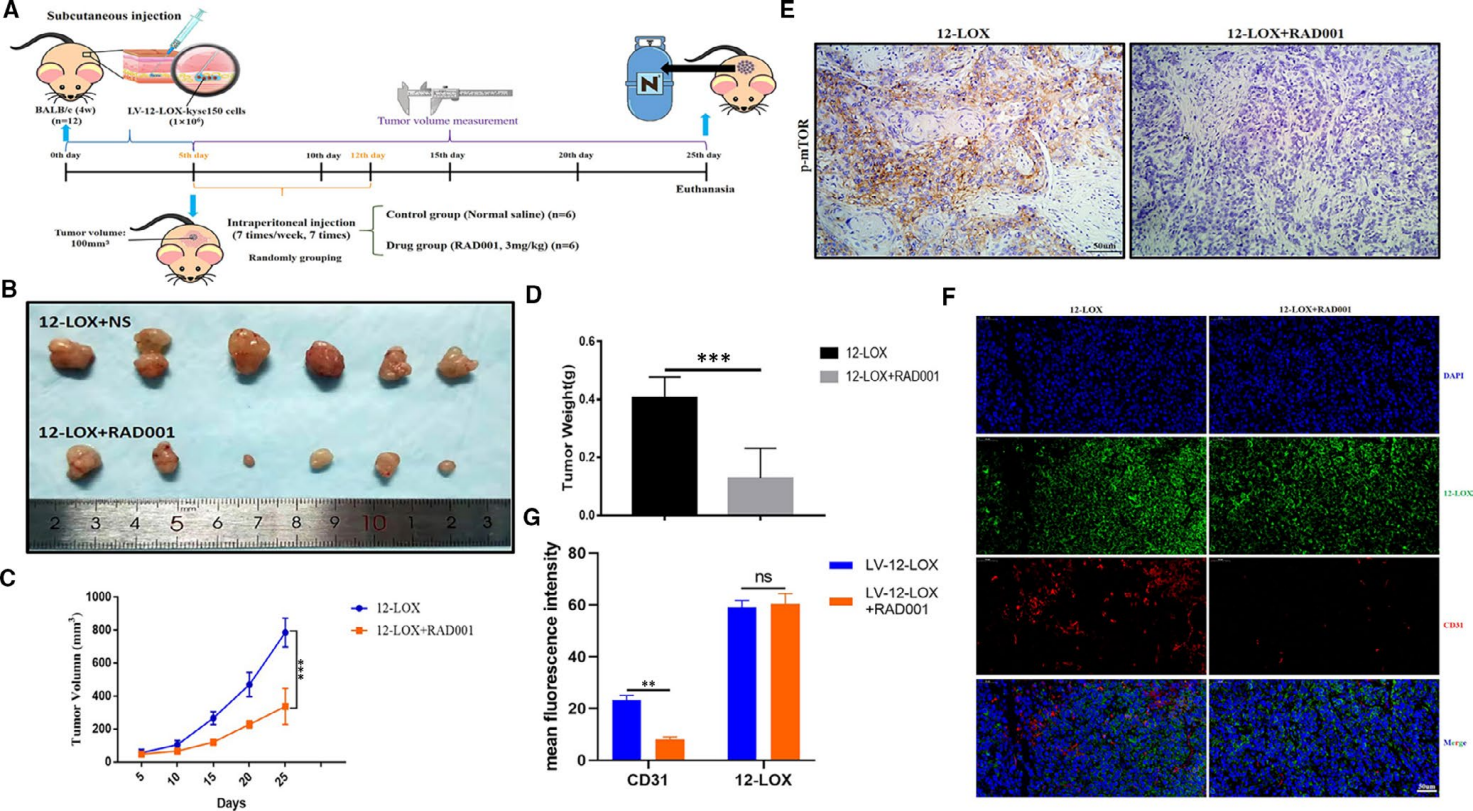
RAD001 reverses the pro‐tumour effect of 12‐LOX in vivo. A, Diagram of intervention in vivo experiments. B, Representative images of subcutaneous tumours with Kyse150 cells in the two different treatment groups indicated. C, Tumour growth curves in nude mice from the two groups. D, Tumour weight of the two groups. E, IHC analyses of p‐mTOR expression were performed using xenografts sections. Scale bar = 50 µm. F, Representative images of IF performed on xenografts treated as indicated with 12‐LOX (green) and CD31 (red) antibodies. Nucleus was labelled with DAPI (blue), and images were merged. Scale bar = 50 µm. G, The expression levels of 12‐LOX and CD31 in the two groups indicated. 12‐LOX, lipoxygenase; IHC, immunohistochemical; IF, immunofluorescence. Data are presented as the mean ± SEM. *P < 0.05; **P < 0.01; ***P < 0.001

Previous in vivo studies demonstrated that RAD001 exerted a significant anti‐tumour effect.^29^, ^36^, ^37^ To further verify the anti‐tumour effect of RAD001 in ESCC, an additional xenograft model with LV‐12‐LOX‐Kyse150 cells was established. As expected, nude mice treated with normal saline (NS) developed large neoplasms reaching an average size of 786mm^3^ at the end of the study period, while mice treated with RAD001 showed a smaller neoplasm size (Figure 6B‐D), confirming that RAD001 significantly inhibited tumour growth.

Subsequently, the inhibitory effects of RAD001 were examined on mTOR signalling by staining tumour sections for p‐mTOR expression (Figure 6E). Following successful inhibition of the pathway, blood vessel formation in tumour tissues was assessed. The results of IF co‐staining with 12‐LOX and CD31 (Figure 6F‐G) indicated that the expression level of 12‐LOX in both groups was high, while the expression level of CD31 in the RAD001‐treated group was lower than that in the saline group. These findings revealed that RAD001 reversed 12‐lipoxygenase‐induced tumour‐promoting effects in vivo by inhibiting angiogenesis of ESCC tumours.

## 4 DISCUSSION

Eicosanoids are known to play an important role in the growth and development of several cancer types. Among various lipoxygenases, 12‐LOX and 12‐HETE are shown to contribute to tumorigenesis in animal models and humans. Overexpression of 12‐LOX protein has been demonstrated in multiple types of cancer, which may play a significant role in carcinogenesis.^38^, ^39^, ^40^ All these studies provided evidence that the 12‐LOX/12‐HETE axis was a potential marker for assessing the progression, metastasis and prognosis of malignant tumours. It is also well known that sustaining proliferative signalling and induction of angiogenesis are two very important hallmarks of malignant cancer.^12^ Our findings demonstrated that 12‐LOX promoted proliferation and angiogenesis of ESCC tumours via the PI3K/AKT/mTOR pathway both in vitro and in vivo. In addition, in all included cases, 73.2% (112 out of 153) of ESCC patients had marked overexpression of 12‐LOX. Moreover, the overall survival time of patients with high expression of 12‐LOX was generally lower than patients who had relatively low expression of 12‐LOX, which was also consistent with the statistical data in the TCGA database. Taken together, the results highlighted that 12‐LOX could effectively promote ESCC, and 12‐LOX might be a potential biological marker for prognostic assessment and treatment of ESCC patients.

The vasculature system in TME is maintained by persistent induction of angiogenic signals^15^ and is characterized by structural abnormalities. Although the abundant vascular system is the key to energy supply and growth of tumour cells, the abnormal vascular structure may cause poor blood flow and elevated blood pressure, leading to hypoxic TME and production of HIF‐1α. Hypoxia will increase VEGF‐A production and the formation of abnormal vessels. The hypoxic regions within the TME may affect the efficacy of radiotherapy or chemotherapy.^35^, ^41^ Furthermore, HIF‐1α is more stable under hypoxic conditions, leading to X‐ray resistance and up‐regulation of genes involved in cell survival.^41^ Meanwhile, emerging studies have reported that hypoxic TME is related to tumour immune tolerance. Hypoxia can stimulate the release of damage‐associated pattern molecules that trigger the immune system's rejection of the tumour. However, the counter‐activation of the tolerance mechanism at tumour hypoxia sites can lead to the tumour's immune escape. It has been found that tumour hypoxia can promote recruitment of regulatory T(Treg) cells by inducing the expression of the chemokine CC‐chemokine ligand 28 (CCL28), which in turn facilitates immune tolerance and angiogenesis.^42^ Hence, certain therapeutic strategies aim to reduce the complexity of tortuous vessels and correct hypoxic TME, which are essential steps in the treatment of malignant tumours.

The main purpose of using anti‐angiogenic drugs in cancer treatment is to destroy tumour angiogenesis. Elevated VEGF‐A levels will increase vascular permeability, leading to vascular leakage, poor blood flow and increased interstitial pressure, thereby exacerbating hypoxia in TME.^35^ Therefore, the use of certain drugs that interfere with the expression or action of VEGF may paradoxically lead to vascular normalization and hypoxic improvement in specific tumour types. VEGFR2 inhibitors can cause the temporary formation of a more regular vascular system.^43^ However, there is a bottleneck in the clinical application of this strategy, given that the induction of vascular normalization is not permanent and that higher exposure to antiangiogenic drugs may increase hypoxia and potentially neutralize the beneficial effects caused by these agents.^44^ Previous studies have explored the possibility of inhibiting carcinogenic signals in cancer cells as another strategy to regulate tumour blood vessels.^44^, ^45^ PI3K/AKT/mTOR signalling pathway inhibitors have a more durable effect on the normalization of tumour blood vessels than of traditional anti‐angiogenic drugs.^44^, ^45^ RAD001, an mTOR pathway inhibitor, has been found to be able to reduce the expression of VEGF and effectively block VEGF‐induced angiogenesis in ESCC. These effects were noted in the present study both in vitro and in vivo, which suggested that RAD001 might play an important role in the treatment of ESCC against the vascular system. It is expected to improve the efficacy of other treatments by normalizing blood vessels and improving hypoxic TME.

As a research hotspot, the PI3K/AKT/mTOR pathway is not only important for signal transduction in normal cells, but also plays an essential role in tumorigenesis and cancer development. This pathway is known to significantly contribute to a variety of cell functions, including proliferation, adhesion, migration, invasion, metabolism, survival and angiogenesis.^21^, ^46^ The tumour‐promoting effect of this pathway in ESCC may involve a complex set of mechanisms. The expression level of mRNA cap‐binding protein eIF‐4E is elevated in multitudinous carcinomas compared with normal tissues and benign lesions. Under normal cell conditions, eIF‐4E combined with inhibitory 4EBPs had little effect on the development of ESCC. When multiple protein members of the PI3K/AKT and RAS‐ERK signalling pathways are phosphorylated, these phosphorylation events can dislodge 4EBPs from eIF‐4E. eIF‐4E in the free‐state can efficiently deliver mRNAs to the eIF‐4F complex, resulting in enhanced transcription of a number of key tumour‐associated proteins, such as cyclin D1, VEGF and matrix metalloprotease 9.^47^ As an inhibitor of the mTOR pathway, RAD001 can inhibit the phosphorylation of 4EBP1, thus preventing the synthesis of tumour‐related proteins. Tang et al have shown that integrinβ4 is a key partner of 12‐LOX and the disruption of integrin β4‐12‐LOX interaction can reduce the pro‐inflammatory and carcinogenic activity of 12‐LOX.^48^ Based on this evidence, we speculated that RAD001 might affect the action of integrin β4 or interfere with integrin β4‐12‐LOX. Therefore, the specific mechanism of RAD001, by which reverses the cancer‐promoting role of 12‐LOX, needs to be further verified.

The present study demonstrated for the first time the role of 12‐LOX in promoting cell proliferation and angiogenesis in ESCC, which provides important information of the role of eicosanoids in malignancy. Concomitantly, the present study demonstrated for the first time that RAD001, an inhibitor of mTOR pathway, played a significant anti‐cancer role in ESCC by targeting the vascular system, which provides a possible target and drug choice for the treatment of this cancer type. However, our analysis did not explore in detail the specific mechanism of the action of RAD001 in ESCC and did not detect the normalization of the vascular architecture, but only detect VEGF expression and tube formation, which was the limitations of the current study.

## ETHICS APPROVAL AND CONSENT TO PARTICIPATE

This study was approved by the Ethics Committee of Qilu Hospital of Shandong University. The above participants agree with the research results described in the article and agree to be responsible for all aspects of the work.

## CONFLICTS OF INTEREST

The authors confirm that there are no conflicts of interest.

## AUTHOR CONTRIBUTION

**Xue Chen:** Conceptualization (lead); Data curation (equal); Methodology (equal); Writing‐original draft (lead); Writing‐review & editing (lead). **Xuan Chen:** Data curation (equal); Methodology (equal); Software (equal). **Xiaozheng Sun:** Formal analysis (equal); Software (equal). **Cong Wang:** Investigation (equal); Validation (equal). **Zhihua Wen:** Formal analysis (equal). **Yufeng Cheng:** Resources (equal); Supervision (equal).

## Supporting information

Fig S1

Fig S2

Fig S3

Fig S4

Table S1

## Data Availability

The data that support the findings of this study are available in the supplementary material of this article. The data set supporting the results of this article is included within the article. The data sets used and/or analysed during the current study are available from the corresponding author on reasonable request.

## References

[1] Ferlay J , Soerjomataram I , Dikshit R , et al. Cancer incidence and mortality worldwide: Sources, methods and major patterns in GLOBOCAN 2012. Int J Cancer. 2015;136:E359‐386. 25220842 10.1002/ijc.29210

[2] Torre LA , Bray F , Siegel RL , Ferlay J , Lortet‐Tieulent J , Jemal A . Global cancer statistics, 2012. CA: Can J Clin. 2015;65(2):87‐108. 10.3322/caac.21262 25651787

[3] Pakzad R , Mohammadian‐Hafshejani A , Khosravi B , et al. The incidence and mortality of esophageal cancer and their relationship to development in Asia. Annals of Translational Medicine. 2016;4(2):29. 26889482 10.3978/j.issn.2305-5839.2016.01.11 PMC4731602

[4] Chen W , Zheng R , Baade PD , et al. Cancer statistics in China, 2015. Ca Cancer J Clin. 2016;66:115‐132. 26808342 10.3322/caac.21338

[5] Pennathur A , Gibson MK , Jobe BA , Luketich JD . Oesophageal carcinoma. The Lancet. 2013;381:400‐412. 10.1016/S0140-6736(12)60643-6 23374478

[6] Alsop BR , Sharma P . Esophageal cancer. Gastroenterol Clin North Am. 2016;45:399‐412. 27546839 10.1016/j.gtc.2016.04.001

[7] Liu Q , Tan W , Che J , et al. 12‐HETE facilitates cell survival by activating the integrin‐linked kinase/NF‐κB pathway in ovarian cancer. Cancer Management and Research. 2018;10:5825‐5838. 30510451 10.2147/CMAR.S180334 PMC6248369

[8] Guo AM , Liu X , Al‐Wahab Z , et al. Role of 12‐lipoxygenase in regulation of ovarian cancer cell proliferation and survival. Cancer Chemother Pharmacol. 2011;68:1273‐1283. 21442472 10.1007/s00280-011-1595-y

[9] Ding XZ , Iversen P , Cluck MW , Knezetic JA , Adrian TE . Lipoxygenase inhibitors abolish proliferation of human pancreatic cancer cells. Biochem Biophys Res Comm. 1999;261:218‐223. 10405349 10.1006/bbrc.1999.1012

[10] Jiang Y , Pan Y , Rhea PR , et al. A sucrose‐enriched diet promotes tumorigenesis in mammary gland in part through the 12‐lipoxygenase pathway. Cancer Res. 2016;76:24‐29. 26729790 10.1158/0008-5472.CAN-14-3432 PMC4703949

[11] Dilly AK , Ekambaram P , Guo Y , et al. Platelet‐type 12‐lipoxygenase induces MMP9 expression and cellular invasion via activation of PI3K/Akt/NF‐kappaB. Int J Cancer. 2013;133:1784‐1791. 23526143 10.1002/ijc.28165 PMC4269488

[12] Honn KV , Tang DG , Gao X , et al. 12‐Lipoxygenases and 12(S)‐HETE: role in cancer metastasis. Cancer Metastasis Rev. 1994;13(3‐4):365‐396. 10.1007/BF00666105 7712597

[13] Lim HJ , Park J , Um JY , Lee SS , Kwak HJ . Zileuton, a 5‐lipoxygenase inhibitor, exerts anti‐angiogenic effect by inducing apoptosis of HUVEC via BK channel activation. Cells. 2019;8:1182. 10.3390/cells8101182 PMC6829222 31575085

[14] Wen Z‐H , Su Y‐C , Lai P‐L , et al. Critical role of arachidonic acid‐activated mTOR signaling in breast carcinogenesis and angiogenesis. Oncogene. 2013;32:160‐170. 22349822 10.1038/onc.2012.47

[15] Hanahan D , Weinberg RA . Hallmarks of cancer: The next generation. Cell. 2011;144:646‐674. 21376230 10.1016/j.cell.2011.02.013

[16] Carmeliet P , Jain RK . Principles and mechanisms of vessel normalization for cancer and other angiogenic diseases. Nat Rev Drug Discov. 2011;10:417‐427. 21629292 10.1038/nrd3455

[17] Gao N , Zhang Z , Jiang B‐H , Shi X . Role of PI3K/AKT/mTOR signaling in the cell cycle progression of human prostate cancer. Biochem Biophys Res Comm. 2003;310:1124‐1132. 14559232 10.1016/j.bbrc.2003.09.132

[18] Ching CB , Hansel DE . Expanding therapeutic targets in bladder cancer: the PI3K/Akt/mTOR pathway. Lab Invest. 2010;90(10):1406‐1414. 10.1038/labinvest.2010.133 20661228

[19] Gao N , Flynn DC , Zhang Z , et al. G1 cell cycle progression and the expression of G1 cyclins are regulated by PI3K/AKT/mTOR/p70S6K1 signaling in human ovarian cancer cells. Am J Physiol Cell Physiol. 2004;287:C281‐C291. 15028555 10.1152/ajpcell.00422.2003

[20] Bader AG , Kang S , Zhao L , Vogt PK . Oncogenic PI3K deregulates transcription and translation. Nat Rev Cancer. 2005;5:921‐929. 16341083 10.1038/nrc1753

[21] Karar J , Maity A . PI3K/AKT/mTOR pathway in angiogenesis. Front Mol Neurosci. 2011;4: 10.3389/fnmol.2011.00051 PMC3228996 22144946

[22] Morgensztern D , McLeod HL . PI3K/Akt/mTOR pathway as a target for cancer therapy. Anticancer Drugs. 2005;16(8):797‐803. 10.1097/01.cad.0000173476.67239.3b 16096426

[23] LoPiccolo J , Blumenthal GM , Bernstein WB , Dennis P . Targeting the PI3K/Akt/mTOR pathway: Effective combinations and clinical considerations. Drug Res Updates. 2008;11(1‐2):32‐50. 10.1016/j.drup.2007.11.003 PMC2442829 18166498

[24] Wullschleger S , Loewith R , Hall MN . TOR signaling in growth and metabolism. Cell. 2006;124:471‐484. 16469695 10.1016/j.cell.2006.01.016

[25] Guertin DA , Sabatini DM . Defining the role of mTOR in cancer. Cancer Cell. 2007;12:9‐22. 17613433 10.1016/j.ccr.2007.05.008

[26] Mathieu L , David M , Cell SJ . mTOR signaling in growth control and disease. Cell. 2012;149(2):274‐293. 10.1016/j.cell.2012.03.017 22500797 PMC3331679

[27] Dancey J . mTOR signaling and drug development in cancer. Nat. Rev Clin Oncol. 2010;7(4):209‐219. 10.1038/nrclinonc.2010.21 20234352

[28] Saran U , Foti M , Dufour JF . Cellular and molecular effects of the mTOR inhibitor everolimus. Clin Sci (Lond). 2015;129:895‐914. 26330617 10.1042/CS20150149

[29] Chao A , Lin C‐Y , Wu R‐C , et al. The combination of everolimus and terameprocol exerts synergistic antiproliferative effects in endometrial cancer: molecular role of insulin‐like growth factor binding protein 2. J Mol Med (Berl). 2018;96:1251‐1266. 30298385 10.1007/s00109-018-1699-5

[30] Avniel‐Polak S , Leibowitz G , Doviner V , Gross DJ , Grozinsky‐Glasberg S . Combining chloroquine with RAD001 inhibits tumor growth in a NEN mouse model. Endocr Relat Cancer. 2018;25(6):677‐686. 10.1530/ERC-18-0121 29636368

[31] Ariaans G , Jalving M , Vries EG , Jong S . Anti‐tumor effects of everolimus and metformin are complementary and glucose‐dependent in breast cancer cells. BMC Cancer. 2017;17:232. 28356082 10.1186/s12885-017-3230-8 PMC5372253

[32] Chen X , Chen X , Liu F , et al. Monocarboxylate transporter 1 is an independent prognostic factor in esophageal squamous cell carcinoma. Oncol Rep. 2019;41:2529‐2539. 30720131 10.3892/or.2019.6992

[33] Moore GY , Pidgeon GP . Cross‐talk between cancer cells and the tumour microenvironment: The role of the 5‐lipoxygenase pathway. Int J Mol Sci. 2017;18. 10.3390/ijms18020236 PMC5343774 28125014

[34] Kim TY , Kim J , Choo HY , Kwon HJ . Inhibition of 5‐lipoxygenase suppresses vascular endothelial growth factor‐induced angiogenesis in endothelial cells. Biochem Biophys Res Commun. 2016;478:1117‐1122. 27530926 10.1016/j.bbrc.2016.08.078

[35] Karar J , Maity A . PI3K/AKT/mTOR pathway in angiogenesis. Front Mol Neurosci. 2011;4:51. 22144946 10.3389/fnmol.2011.00051 PMC3228996

[36] Alshaker H , Wang QI , Böhler T , et al. Combination of RAD001 (everolimus) and docetaxel reduces prostate and breast cancer cell VEGF production and tumour vascularisation independently of sphingosine‐kinase‐1. Sci Rep. 2017;7:3493. 28615679 10.1038/s41598-017-03728-3 PMC5471177

[37] Hortobagyi GN , Chen D , Piccart M , et al. Correlative analysis of genetic alterations and everolimus benefit in hormone receptor‐positive, human epidermal growth factor receptor 2‐negative advanced breast cancer: Results from BOLERO‐2. J Clin Oncol. 2016;34:419‐426. 26503204 10.1200/JCO.2014.60.1971 PMC5070556

[38] Ashok‐kumar DP . Platelet‐type 12‐lipoxygenase induces MMP9 expression and cellular invasion via activation of PI3K/Akt/NF‐κB. 10.1002/ijc.28165 PMC4269488 23526143

[39] Singh AK , Kant S , Parshad R , Banerjee N , Dey S . Evaluation of human LOX‐12 as a serum marker for breast cancer. Biochem Biophys Res Commun. 2011;414:304‐308. 21945939 10.1016/j.bbrc.2011.09.044

[40] Kerjaschki D , Bago‐Horvath Z , Rudas M , et al. Lipoxygenase mediates invasion of intrametastatic lymphatic vessels and propagates lymph node metastasis of human mammary carcinoma xenografts in mouse. Journal of Clinical Investigation. 2011;121(5):2000‐2012. 10.1172/JCI44751 PMC3083794 21540548

[41] Barker HE , Paget JT , Khan AA , Harrington KJ . The tumour microenvironment after radiotherapy: Mechanisms of resistance and recurrence. Nat Rev Cancer. 2015;15:409‐425. 26105538 10.1038/nrc3958 PMC4896389

[42] Facciabene A , Peng X , Hagemann IS , et al. Tumour hypoxia promotes tolerance and angiogenesis via CCL28 and T(reg) cells. Nature. 2011;475:226‐230. 21753853 10.1038/nature10169

[43] Winkler F , Kozin SV , Tong RT , et al. Kinetics of vascular normalization by VEGFR2 blockade governs brain tumor response to radiationRole of oxygenation, angiopoietin‐1, and matrix metalloproteinases. Cancer Cell. 2004;6(6):553‐563. 10.1016/S1535-6108(04)00305-8 15607960

[44] Fokas E , Im JH , Hill S , et al. Dual inhibition of the PI3K/mTOR pathway increases tumor radiosensitivity by normalizing tumor vasculature. Cancer Res. 2012;72:239‐248. 22108822 10.1158/0008-5472.CAN-11-2263

[45] Qayum N , Muschel RJ , Im JH , et al. Tumor vascular changes mediated by inhibition of oncogenic signaling. Can Res. 2009;69(15):6347‐6354. 10.1158/0008-5472.CAN-09-0657 PMC2825046 19622766

[46] Bader AG , Kang S , Zhao L , Oncogenic VPK . Oncogenic PI3K deregulates transcription and translation. Nat Rev Cancer. 2005;5(12):921‐929. 10.1038/nrc1753 16341083

[47] De Benedetti A , Graff JRJO . eIF‐4E expression and its role in malignancies and metastases. Oncogene. 2004;23(18):3189‐3199. 10.1038/sj.onc.1207545 15094768

[48] Tang K , Cai Y , Joshi S , et al. Convergence of eicosanoid and integrin biology: 12‐lipoxygenase seeks a partner. Mol Cancer. 2015;14:111. 26037302 10.1186/s12943-015-0382-5 PMC4453211

