# Supplementary figures and images for: RAD001 targeted HUVECs reverses 12‐lipoxygenase‐induced angiogenesis in oesophageal squamous cell carcinoma
Source: J Cell Mol Med. 2021 Jun 13;25(14):6936–47. doi: 10.1111/jcmm.16705 (PMC8278093; doi:10.1111/jcmm.16705)

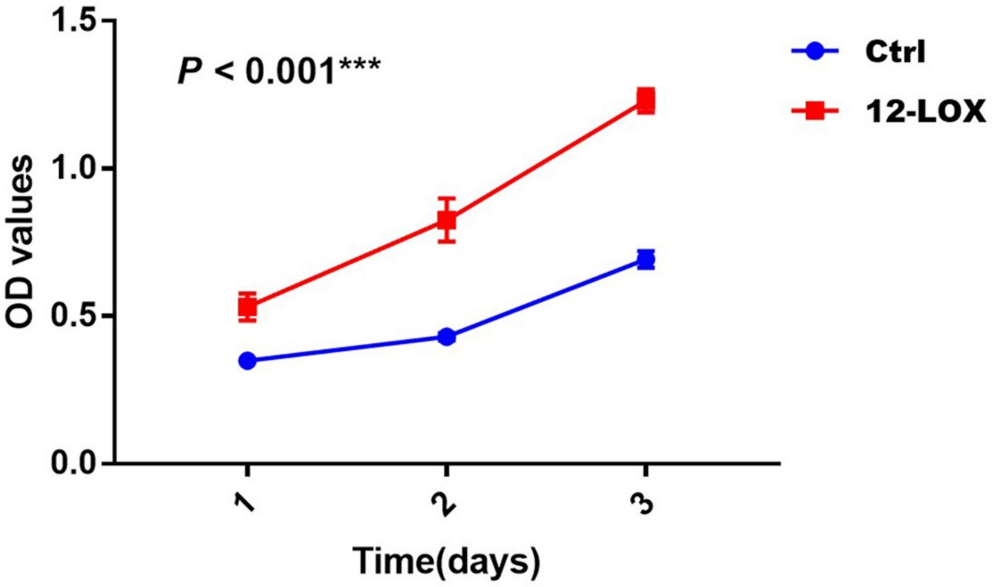

Supplement: Supplementary file 1 — Fig S1 [file JCMM-25-6936-s004.tif]

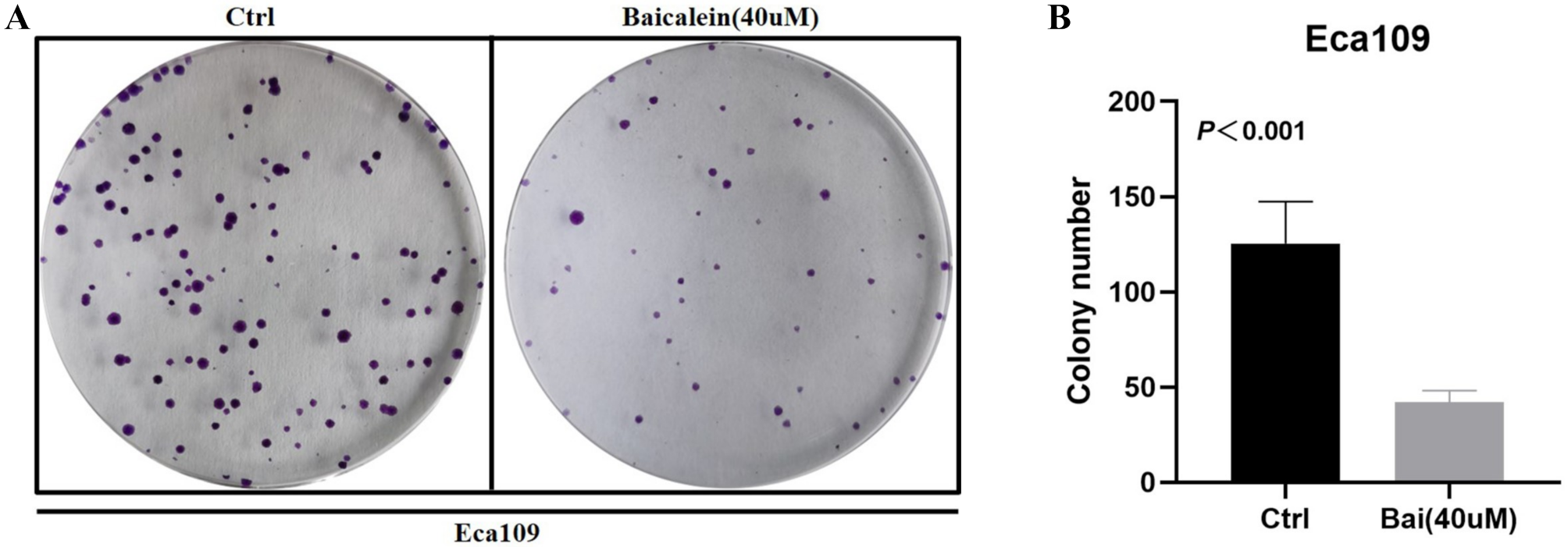

Supplement: Supplementary file 2 — Fig S2 [file JCMM-25-6936-s001.tif]

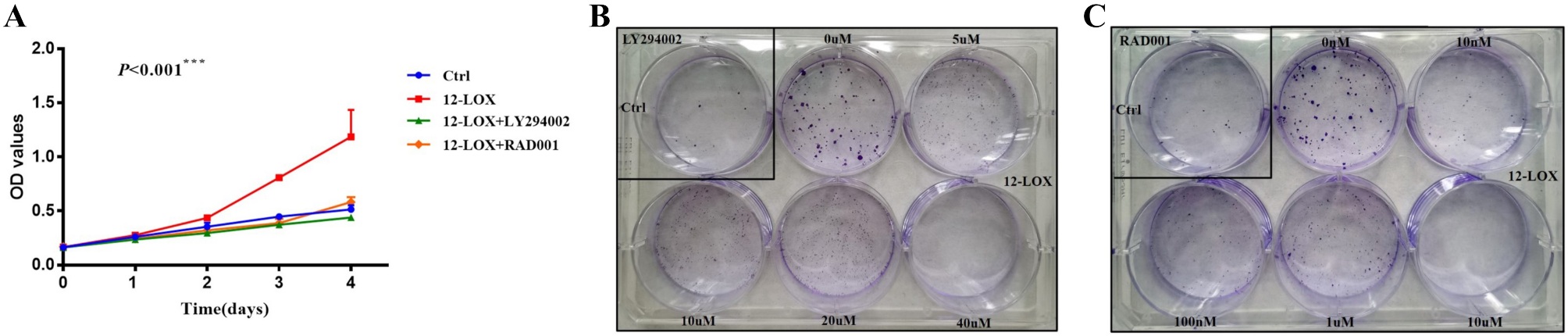

Supplement: Supplementary file 3 — Fig S3 [file JCMM-25-6936-s002.tif]

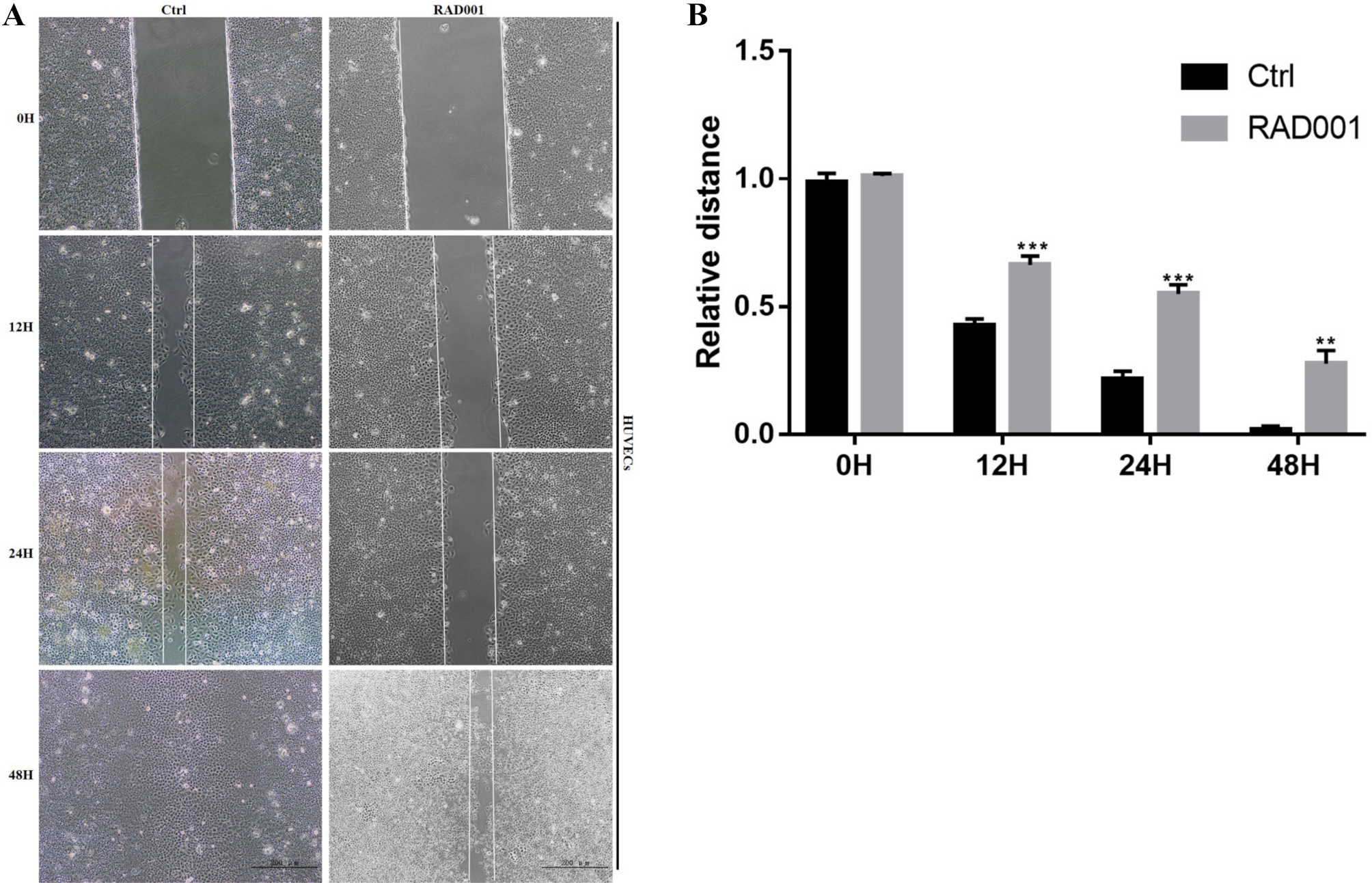

Supplement: Supplementary file 4 — Fig S4 [file JCMM-25-6936-s005.tif]
