# Supplementary material for: RAD001 targeted HUVECs reverses 12‐lipoxygenase‐induced angiogenesis in oesophageal squamous cell carcinoma
Source: J Cell Mol Med. 2021 Jun 13;25(14):6936–47. doi: 10.1111/jcmm.16705 (PMC8278093; doi:10.1111/jcmm.16705)
Supplement: Supplementary file 5 — Table S1 [file JCMM-25-6936-s003.docx]

**Supplementary Table S1** Baseline characteristics of the 153 esophageal squamous cell carcinoma patients

| **Characteristics** | **Value, n (%)** |
| --- | --- |
| Sex |  |
| Female | 74(48.3) |
| Male | 79(51.6) |
| Age |  |
| >65 | 88(57.5) |
| ≤65 | 65(42.4) |
| Smoking |  |
| Yes | 88(57.5) |
| No | 65(42.4) |
| Drinking |  |
| Yes | 83(54.2) |
| No | 70(45.7) |
| Differentiation degree |  |
| Well | 46(30.0) |
| Middle | 44(28.7) |
| Poor | 63(41.1) |
| T stage |  |
| T1 | 17(11.1) |
| T2 | 58(37.9) |
| T3 | 51(33.3) |
| T4 | 27(17.6) |
| N stage |  |
| N0 | 59(38.5) |
| N1-3 | 94(61.5) |
| pTNM stage |  |
| I | 24(15.6) |
| II | 52(33.9) |
| III | 77(50.3) |
| 12-LOX expression |  |
| Low | 41(26.7) |
| Over | 112(73.2) |

N stage, lymph node metastasis; pTNM, pathological TNM; T stage, invasion depth.
